# Efficacy of c-Met inhibitor for advanced prostate cancer

**DOI:** 10.1186/1471-2407-10-556

**Published:** 2010-10-14

**Authors:** William H Tu, Chunfang Zhu, Curtis Clark, James G Christensen, Zijie Sun

**Affiliations:** 1 Department of Urology and Department of Genetics, Stanford University School of Medicine, Stanford, CA 94305-5328, USA; 2 Department of Cancer Research, Pfizer Global Research and Development, La Jolla Laboratories, La Jolla, CA 92121, USA

## Abstract

**Background:**

Aberrant expression of HGF/SF and its receptor, c-Met, often correlates with advanced prostate cancer. Our previous study showed that expression of c-Met in prostate cancer cells was increased after attenuation of androgen receptor (AR) signalling. This suggested that current androgen ablation therapy for prostate cancer activates c-Met expression and may contribute to development of more aggressive, castration resistant prostate cancer (CRPC). Therefore, we directly assessed the efficacy of c-Met inhibition during androgen ablation on the growth and progression of prostate cancer.

**Methods:**

We tested two c-Met small molecule inhibitors, PHA-665752 and PF-2341066, for anti-proliferative activity by MTS assay and cell proliferation assay on human prostate cancer cell lines with different levels of androgen sensitivity. We also used renal subcapsular and castrated orthotopic xenograft mouse models to assess the effect of the inhibitors on prostate tumor formation and progression.

**Results:**

We demonstrated a dose-dependent inhibitory effect of PHA-665752 and PF-2341066 on the proliferation of human prostate cancer cells and the phosphorylation of c-Met. The effect on cell proliferation was stronger in androgen insensitive cells. The c-Met inhibitor, PF-2341066, significantly reduced growth of prostate tumor cells in the renal subcapsular mouse model and the castrated orthotopic mouse model. The effect on cell proliferation was greater following castration.

**Conclusions:**

The c-Met inhibitors demonstrated anti-proliferative efficacy when combined with androgen ablation therapy for advanced prostate cancer.

## Background

Prostate cancer is the most common malignancy in men in the United States [1]. While the mortality of prostate cancer has been slightly reduced recently, it still contributes to 30,000 deaths annually with the majority from castration resistant prostate cancer (CRPC) [2]. The androgen-signaling pathway, mediated mostly through the androgen receptor (AR), plays a critical role in the regulation of prostate cancer cell growth and survival [3,4]. Androgen deprivation is the standard therapy for advanced prostate cancer [5]. However, within two to three years after initiating therapy, most patients relapse with a more aggressive form of prostate cancer, termed CRPC, for which there is no effective treatment.

The c-Met receptor tyrosine kinase (RTK) was originally discovered as an oncoprotein and has been implicated in the proliferation and progression of a wide variety of human malignancies, including prostate cancer [6-9]. High c-Met expression is observed in late stages and metastases of prostate cancer [8,10]. Additionally, an inverse correlation between the expression of AR and c-Met has been observed in prostate epithelium and prostate cancer cell lines [8,10]. Recently, we demonstrated that AR suppressed c-Met transcription and that increased c-Met expression was induced by removal of androgens in prostate cancer cells [11]. These data elucidated a biological role for AR in c-Met transcription that may directly contribute to the pathogenesis of CRPC. While the current androgen deprivation therapy represses the expression of growth promoting genes that are activated by the AR, it may also attenuate the suppressive role of AR on c-Met expression and contribute to tumor progression.

In this study, we directly assessed the inhibition of c-Met signalling pathway in prostate cancer cell growth and tumor formation and progression using two c-Met inhibitors, PHA-665752 and PF-2341066. These inhibitors have shown potency and specificity for inhibiting c-Met activation in a variety of human tumor cells [12-16]. We first tested the anti-proliferative effect of these inhibitors on a variety of human prostate cancer cell lines. We then examined the effect of PF-2341066 in inhibiting the proliferation of LNCaP tumors in renal subcapsular mouse models. Finally, we assessed the effect of co-inhibition of c-Met and androgen signalling pathways in prostate cancer progression using PF-2341066 and castration in an orthotopic xenograft model. Through these *in vitro *and *in vivo *experimental approaches, we explored a future therapeutic strategy of combining c-Met inhibitors with standard androgen ablation therapy for advanced prostate cancer.

## Methods

### Cell culture

Human prostate cancer cell line LAPC4 was maintained in RPMI phenol-red free (Invitrogen, Carisbad, CA) supplemented with 5% fetal bovine serum (FBS, HyClone, Denver, CO) [17-19]. Human prostate cancer cell line CWR22Rv1 was maintained in RPMI (Invitrogen) supplemented with 5% FBS and obtained from the American Tissue Culture Collection (ATCC) (CRL-2505). Human prostate cancer cell lines LNCaP, LNCaP C4-2, and LNCaP C4-2B were maintained in T medium (Invitrogen) supplemented with 5% FBS [18]. Human prostate cancer cell lines DU-145 and PC-3 were maintained in DMEM supplemented with 5% FBS and obtained from ATCC (HTB-81, CRL-1435).

### Cell proliferation and colony formation assays

Five hundred cells per well were seeded in triplicate in 96-well plates in suitable media mentioned above. Appropriate controls or designated concentrations of PHA-665752 or PF-2341066 dissolved in DMSO were added to each well after 4 hours, and cells were then incubated for up to 8 days. Cell proliferation assays were carried out using the MTS (3-(4,5-dimethylthiazol-2-yl)-5-(3-carboxymethoxyphenyl)-2-(4-sulfophenyl)-2H-tetrazolium) tetrazolium kit (Promega, Madison, WI) as suggested by the manufacturer. For colony formation assay, 50 cells per well were plated in quadruplicate in 6-well plates for 24 hr, and then 5 μM of PHA-665752 or PF-2341066 dissolved in DMSO was added to each well. Cells were then grown for 10 days then fixed and stained with crystal violet in methanol. Each visible colony (> 50 cells) was manually counted.

### C-Met inhibitors

PHA-665752 [(2R)-1-[[5-[(Z)-[5-[[(2,6-Dichlorophenyl)methyl]sulfony l]-1,2-dihydro-2-oxo-3H-indol-3-ylidene]methyl]-2,4-dim ethyl-1H-pyrrol-3-yl]carbonyl]-2-(1-pyrrolidinylmethyl) pyrrolidine] was acquired from Tocris Bioscience, Missouri 12. PF-2341066 [(R)-3-[1-(2,6-dichloro-3-fluoro-phenyl)-ethoxy]-5-(1-piperidin-4-yl-1*H*-pyrazol-4-yl)-pyridin-2-ylamine] was provided by Dr. James Christensen in Pfizer, La Jolla Laboratories [14].

### Western blotting

To prepare the whole-cell lysate, cells were washed with cold PBS and resuspended in radioimmunoprecipitation assay (RIPA) buffer, 1% NP40, 0.1% SDS, 50 mM NaF, 0.2 mM Na_3_VO_4_, 0.5 mM DTT, 150 mM NaCl, 2 mM EDTA, 10 mM sodium phosphate buffer (pH 7.2). Protein samples boiled in SDS-sample buffer were resolved on a 6% SDS-PAGE, transferred onto a nitrocellulose membrane, and then blotted with a polyclonal anti-c-Met antibody (Santa Cruz Biotechnology, Santa Cruz, CA), monoclonal anti-phospho-c-Met antibody (Cell Signaling Technology, Danvers, MA), or monoclonal anti-tubulin antibody (Thermo Scientific, Fremont, CA). Blots were detected using the enhanced chemiluminescence kit (Amersham, Arlington Heights, IL).

### Animal studies

All animal studies performed in this study conformed to the Helsinki Declaration and to local legislation. The research was approved by the ethics committee of the Administrative Panel on Laboratory Animal Care at Stanford University. Male nonobese diabetic-severe combined immunodeficient (NOD-SCID) mice (6 weeks old) were obtained from Jackson Laboratory (Bar Harbor, ME), and maintained and cared in accordance with the Guide for the Care and Use of Laboratory Animals.

LNCaP C4-2 cells were suspended in unpolymerized rat tail collagen to make collagen grafts (5 × 10^5 ^cells per graft). Two grafts were implanted under the renal capsule of each male NOD-SCID mouse that was then subcutaneously implanted with a testosterone pellet (10 mg/mouse) [20,21]. At 1 week post-grafting, one group of randomly chosen mice was started on daily treatment with PF-2341066 at 50 mg/kg given in water by oral gavage for 3 weeks. At 4 weeks post-grafting, these animals were sacrificed with the control group on water only. The grafts from the mice on water (n = 8) or PF-2341066 (n = 10) were harvested, weighed, and fixed for pathological analyses. Significant differences in graft weight between the treated versus the control groups were determined using Student's *t *test.

For the orthotopic xenograft mouse model, LNCaP cells (1 × 10^6^) were suspended in 50 μl of unpolymerized rat tail collagen and injected into the dorsal prostate of male NOD-SCID mice, supplemented with testosterone pellets (10 mg/mouse) [22-25]. At five weeks post-implantation, a subset of mice (n = 4) was sacrificed to collect prostates for histopathologic analyses as a pre-castration group. The remaining mice were castrated and the testosterone pellets were removed. At one week post-castration, animals were either given daily treatment with PF-2341066 at 50 mg/kg in water (n = 7) or water only as controls (n = 6) by oral gavage for 3 weeks. Then, the mice were sacrificed and the prostate and pelvic lymph nodes were harvested and fixed for pathological analyses.

### Immunohistochemistry

Tissues were fixed in 10% neutral buffered formalin, processed, cut at 4 μm intervals, dewaxed, and hydrated as described previously [11]. Endogenous peroxidase activity was blocked with 0.5% hydrogen peroxide and then incubated with the blocking solution (ImmunoVision Technology, Springdale, AR). The adjacent sections were incubated with primary antibody or nonimmune mouse or rabbit IgG overnight at 4°C (Zymed Corp, So. San Francisco, CA). The mouse anti-human AR (Santa Cruz Biotechnology), rabbit anti-human PSA (DAKO, Carpinteria, CA), or rabbit anti-human Ki67 (Leica Microsystems, Bannockburn, IL) was used at dilutions of 1:300, 1:3000, or 1:3000, respectively, in PBS with 1% goat serum. Following incubation, sections were washed, incubated with a biotinylated goat anti-rabbit or anti-mouse antibody (Vector Laboratories, Burlingame, CA) at a dilution of 1:500, and then incubated with avidin-biotin complex (Vector Laboratories) at a dilution of 1:500. All sections used for immunohistochemistry were lightly counterstained with 5% (w/v) Harris hematoxylin. Stained sections were analyzed using an Olympus BX51 light microscope and DP70 camera with a 20× objective lens. Quantitative Ki67 index was determined by counting the number of Ki-67-positive cells relative to the number of total cells in more than five fields of two representative sections from each sample via light microscopy.

## Results

### C-Met inhibitors decrease proliferation of AR negative prostate cancer cells

**Figure 1:**
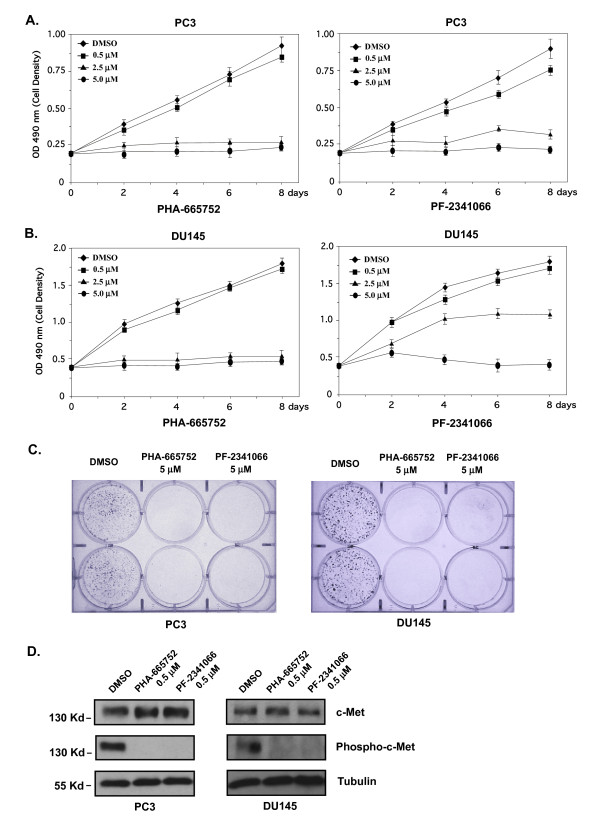
**c-Met inhibitors repressed cell growth and c-Met receptor phosphorylation in PC-3 and DU-145 prostate cancer cells**. (**A**) PC-3 cells were cultured with DMEM supplemented with 5% FBS. The c-Met inhibitor, PHA-665752 or PF-2341066, dissolved in DMSO was added to the growth media at the indicated concentration. Cells were harvested and analyzed by the MTS assay at indicated time-points. Absorbance at 490 nm was measured in triplicate samples. The data represent the mean ± S.D. of three independent experiments. (**B**) The similar MTS assays were carried out in DU-145 cells. (**C**) Approximately 50 PC-3 or DU-145 cells per well were plated in quadruplicate in 6-well plates for 24 hr, and then PHA-665752 or PF-2341066 was added to cells. Cells were allowed to grow for 10 days then fixed and stained with crystal violet. (**D**) PC-3 or DU-145 cells were cultured with 5% FBS-DMEM and were then exposed for 3 hours to DMSO alone, 0.5 μM of PHA-665752, or 0.5 μM of PF-2341066 dissolved in DMSO. Whole cell lysates were prepared from the above cells for immunoblotting with c-Met or phosphorylated c-Met antibody, as well as the β-tubulin antibody as a loading control.

PC-3 and DU-145 are two frequently used human prostate cancer cell lines that have shown strong c-Met expression [7,8,10,26]. We examined the effect of two small molecule inhibitors for c-Met, PHA-665752 and PF-2341066, on the growth of PC-3 and DU-145 cells by MTS assays. As shown in Figures 1A, both PHA-665752 and PF-2341066 fully inhibited the growth of PC-3 cells at 2.5 μM, which is consistent with earlier pharmacokinetic and efficacy studies on other human cancer cells lines [12-16]. The effect was sustained over time during the course of the experiments. An almost identical effect of PHA-665752 was observed on DU-145 cells during the course of the experiments though PF-2341066 showed a slightly weaker inhibition at 2.5 μM (Fig. 1B). We then assessed the inhibitory effects of PHA-665752 and PF-2341066 using colony formation assay. Approximately 50 tumor cells were seeded in each well in the presence of 5 μM PHA-665752 or PF-2341066. In both PC-3 and DU-145 cells, there was no visible colony formation in the presence of either inhibitor after 8 days (Fig. 1C). The above data demonstrated an inhibitory effect of the two c-Met inhibitors on the proliferation of prostate cancer cells.

As described previously, PHA-665752 and PF-2341066 were one class of selective, ATP-competitive c-Met inhibitors defined by the indolin-2-one core structure [12,14]. They inhibit tyrosine phosphorylation of c-Met to block activation of the receptor for downstream signaling. To directly assess the role of these inhibitors on c-Met phosphorylation, we cultured PC-3 and DU-145 cells in the presence of 0.5 μM of PHA-665752 or PF-2341066. After 3 hr treatment, whole cell lysates were isolated and analyzed by Western blotting. While similar levels of the c-Met protein appeared in both inhibitor treated and untreated samples from PC-3 and DU-145 cells, phosphorylated c-Met protein was not detected in the samples treated with the inhibitors (Fig. 1D). The results demonstrated that both PHA-665752 and PF-2341066 inhibit the phosphorylation of c-Met in prostate cancer cells.

### C-Met inhibitors reduce proliferation of AR positive prostate cancer cells

**Figure 2:**
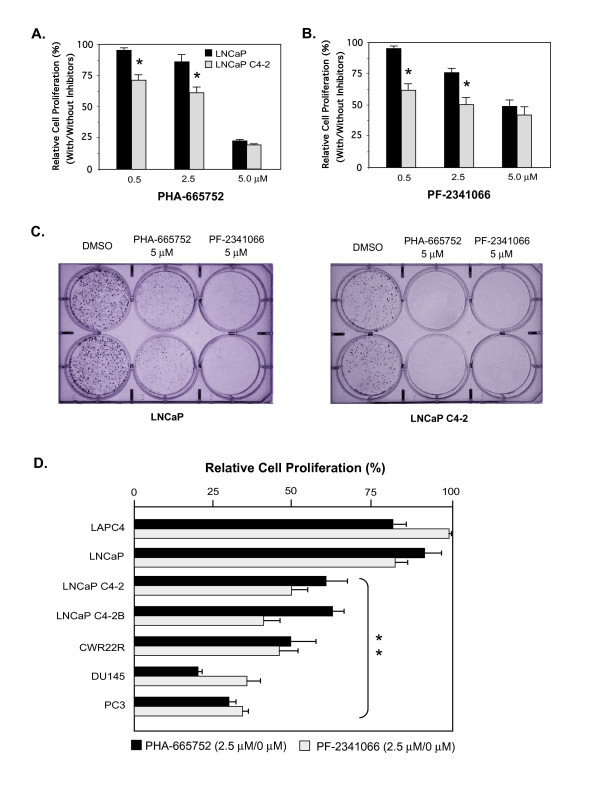
**c-Met inhibitors suppressed growth of AR positive prostate cancer cells, especially androgen insensitive type**. (**A**) Both LNCaP and LNCaP C4-2 cells were analyzed in MTS assay in the presence of PHA-665752. The average relative cell proliferation after 8 days as a percentage of proliferation in the DMSO vehicle control ± SE of triplicate samples was plotted, and the significant difference was observed as *P < 0.01 (Student's *t *test). (**B**) The effect of PF-2341066 on LNCaP and LNCaP C4-2B cells was tested and analyzed similarly as (**A**). The significant difference was shown, *P < 0.01 (Student's *t *test). (**C**) Approximately 50 LNCaP or LNCaP C4-2 cells per well were plated in quadruplicate in 6-well plates for 24 hr, and then 5 μM of PHA-665752 or PF-2341066 was added. Cells were fixed and stained with crystal violet in methanol at day 10. (**D**) The relative anti-proliferative effect of PHA-665752 or PF-2341066 at 2.5 μM on different prostate cancer cells was compared. A significant difference, **P < 0.01 (Student's *t *test), between two AR positive cell lines, LNCaP and LAPC4, and other AR negative and androgen insensitive cells was observed.

AR is still expressed in most CRPC and plays a critical role during disease progression [3,4,27,28]. To extend our study to this clinical scenario, we next examined the effect of the c-Met inhibitors in LNCaP, an AR positive androgen sensitive prostate cancer line, and LNCaP C4-2, an androgen-insensitive subline, derived from LNCaP cells grown in a castrated host [18]. The effect of c-Met inhibitors on LNCaP and its subline was assessed in MTS assay. Both PHA-665752 (Fig. 2A) and PF-2341066 (Fig. 2B) showed a dose dependent inhibition in the growth of LNCaP and LNCaP C4-2 cells. Interestingly, C4-2 cells showed a greater response to PHA-665752 and PF-2341066 mediated inhibition than LNCaP cells, supporting the previous observation that androgen insensitive cells have higher expression of c-Met [7,8,10]. Similar inhibitory effects of PHA-665752 and PF-2341066 on LNCaP and C4-2 cells were also observed in colony formation assays (Fig. 2C). The above data demonstrated a suppressive effect of the c-Met inhibitors on the growth of AR positive prostate cancer cells.

Next, we further assessed the effect of the c-Met inhibitors on other AR positive prostate cancer cell lines, including LAPC4, LNCaP C4-2B, and CWR22Rv1 [17-19]. In MTS assays, all of the above cell lines showed dose dependent growth inhibition in the presence of PHA-665752 or PF-2341066 compared to DMSO vehicle control. Analysis of the relative inhibition rates of PHA-665752 or PF-2341066 at 2.5 μM on prostate cancer cells showed an inverse association between drug responsiveness and androgen sensitivity status (Fig. 2D). Two AR negative cell lines, PC-3 and DU-145, were most responsive to the c-Met inhibitors followed by the androgen insensitive cells, LNCaP C4-2, C4-2B, and CWR22Rv1, whereas the androgen sensitive LNCaP and LAPC4 cells showed much less responsiveness. Overall, the data indicated that c-Met inhibition had greater anti-proliferative efficacy as prostate cancer cells became more androgen resistant.

### C-Met inhibitor, PF-2341066, suppresses growth of AR positive prostate tumors in mice

Next, we assessed the potential of c-Met inhibitor in tumor formation of AR positive, androgen insensitive cells using an *in vivo *mouse model. Specifically, we tested the efficacy of PF-2341066 since it was more clinically relevant as an orally bioavailable and water-soluble drug [14]. The subcapsular renal tumor model was chosen in this set of experiments for its high implant survival rate and ease in separating tumor from host tissue for accurate measurements of tumor growth [11,20,21].

**Figure 3:**
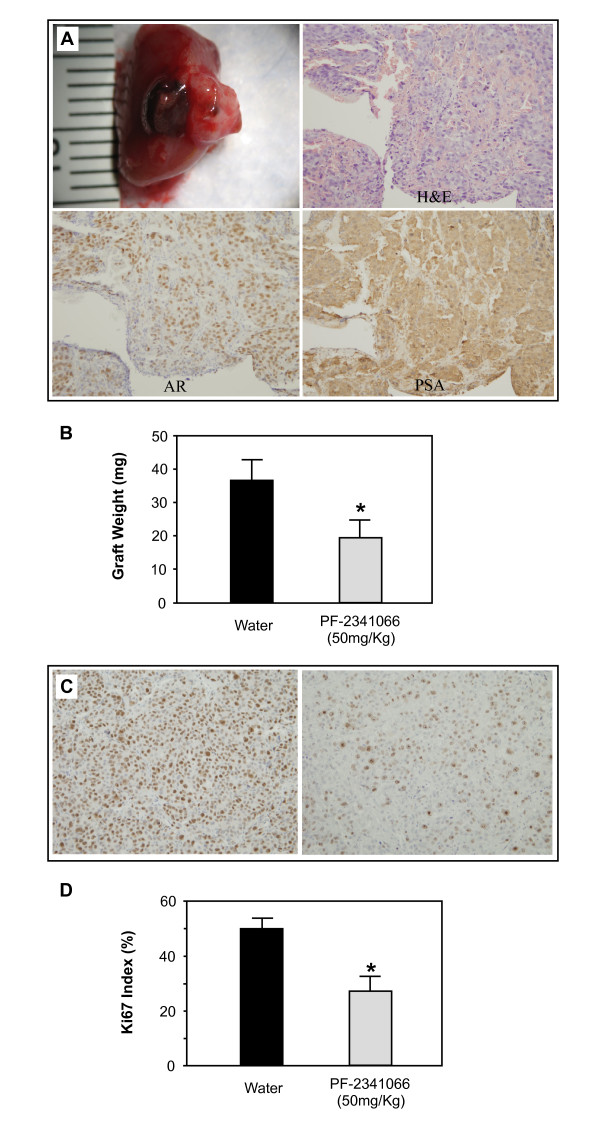
**c-Met inhibitor decreased proliferation of androgen receptor positive cells *in vivo***. LNCaP C4-2 cells were implanted into the renal capsule of male SCID mice supplemented with testosterone as described in the "Materials and Methods". At day 28, mice were sacrificed and the grafted tumor mass was identified. (**A**) Representative tumor tissue stained with hematoxylin and eosin, or analyzed by immunostaining with human AR or PSA antibody, respectively. (**B**) Tumor tissues were harvested and weighed from mice given water or daily oral gavage of PF-2341066 at 50 mg/kg. The difference of weight was analyzed by Student's *t *test (*P < 0.01). (**C**) Representative tumor tissue isolated from mice given water (left) or daily oral gavage of PF-2341066 at 50 mg/kg (right) and analyzed by immunostaining with Ki67 antibody. (**D**) Positive cells with Ki67 antibody were counted in more than 5 different fields, and the Ki67 index was measured as the number of the positive Ki67 cells relative to the number of total cells in two representative sections. There was a difference between the control and treated mice, *P < 0.01 (Student's *t *test).

AR positive LNCaP C4-2 cells were grafted under the kidney capsule of 8 week-old male SCID mice as reported previously [11,21]. One week after grafting, mice were divided into groups that received either daily oral gavage with 50 mg/kg of PF-2341066 (n = 10) or water only (n = 8) for 3 weeks. At the end of 4 weeks, mice were sacrificed and tumor grafts were harvested, weighed, and fixed in formalin for further pathological analysis. LNCaP C4-2 tumor grafts were observed under the capsule of kidneys as presented in Figure 3A. Histologic evaluation of the graft tissues showed a mass of LNCaP C4-2 tumor cells. Tumor cells showed positive nuclear immunostaining for human AR and cytoplasmic staining for human PSA, confirming the tumor origin as LNCaP C4-2 cells. Analysis of LNCaP C4-2 tumor grafts showed that the weights of tumors in mice treated with PF-2341066 were significantly less than the ones in water vehicle controls (Fig. 3B).

To determine if the difference in tumor growth was associated with an anti-proliferative effect, immunostaining for the proliferation marker Ki67 was performed on the above graft tumor tissues isolated from either the control group (left panel, Fig. 3C) or PF-2341066 treated mice (right panel, Fig. 3C). The relative proliferative index as a percent of Ki67 positive cells was significantly reduced in the tissues isolated from mice treated with PF-2341066 (Fig. 3D). These data demonstrated that the c-Met inhibitor suppresses the growth of AR positive, androgen insensitive prostate cancer cells in xenograft mouse models, implying a potential use of the inhibitor in repressing the progression of prostate cancer cells.

### C-Met inhibitor, PF-2341066, represses prostate cancer cell growth during the progression of AR positive prostate tumors in castrated mice

Previous studies have shown that the orthotopic prostate cancer mouse model can mimic the course of human prostate cancer progression after castration [22-25]. In this model, AR positive and androgen sensitive LNCaP cells were implanted into mouse prostates. After tumor development, mice were castrated. LNCaP cells were initially inhibited after castration, then regained growth ability and developed castration resistant disease with potential for metastasis. Thus, we used this model to directly test whether co-targeting androgen and c-Met signaling pathways could effectively inhibit tumor cell growth.

**Figure 4:**
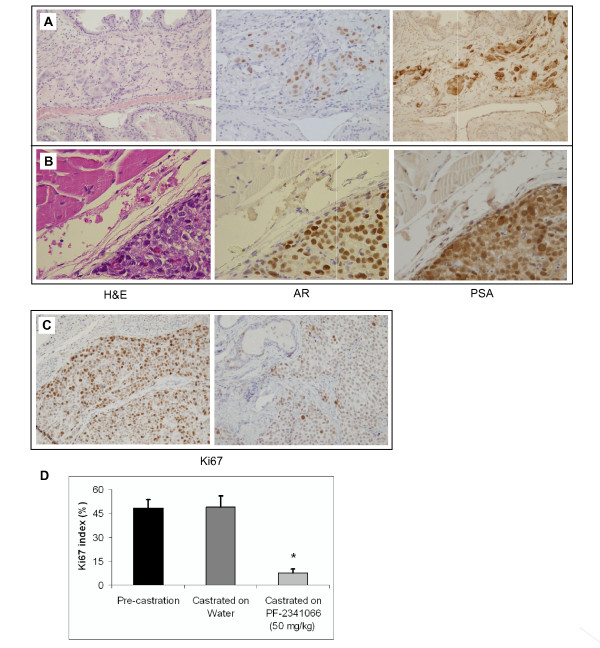
**c-Met inhibitor combined with castration reduced proliferation of castration resistant prostate cancer cells in vivo**. LNCaP cells were orthotopically implanted into the prostates of male SCID mice supplemented with testosterone (see "Materials and Methods"). (**A**) Prostate tissue isolated from a representative non-castrated 11 week-old male SCID mouse and analyzed with hematoxylin and eosin staining and immunohistochemistry with the human AR antibody or PSA antibody. (**B**) Prostate tissue stained with hematoxylin and eosin, human AR antibody, and PSA antibody that was isolated from a representative 15 week-old male SCID mouse that had been castrated at 11 weeks-old. (**C**) Representative prostate tissue analyzed with Ki67 antibody that was isolated from castrated animals that were given either water (left) or PF-2341066 at 50 mg/kg (right) for 4 weeks post-castration. (**D**) Ki67 index was measured in pre-castration, control castrated mice on water, and castrated mice treated with PF-2341066. There was a significant difference between control and treated mice, *P < 0.01 (Student's t test).

Approximately 1 × 10^6 ^LNCaP cells were orthotopically implanted into the dorsal prostate of each 8 week-old male SCID mouse that was then supplemented with testosterone. At five weeks post-implantation, a subset of mice (n = 4) as a pre-castration group was sacrificed to harvest the prostates for evidence of tumor formation after orthotopic implantation and to determine a baseline cancer cell proliferation rate. Hematoxylin and eosin staining showed clusters of large cells with nuclear atypia adjacent to normal mouse prostate glands in 3 of 4 mice (Fig. 4A). Positive immunostaining of serial sections for human AR (middle, Fig. 4A) and PSA (right, Fig. 4A) demonstrated that LNCaP tumors formed in the mouse prostate after orthotopic implantation.

To study the progression of the tumors, the remaining mice were castrated. At one-week post-castration, one subset of castrated mice (n = 7) was given daily oral gavage with 50 mg/kg of PF-2341066 in water for 3 weeks. Another group of mice (n = 6) was given water only for 3 weeks as controls. Mice were sacrificed and carefully examined at the end of the fourth week of castration. In the control group, LNCaP tumors appeared in 4 of 6 mice. Large tumor cells with nuclear atypia were clearly observed in the samples stained with hematoxylin and eosin (left, Fig. 4B). Identity of LNCaP cells was further confirmed by positive immunostaining for human AR (middle, Fig. 4B) and PSA (right, Fig. 4B). In addition, a pelvic lymph node metastasis of LNCaP cells was identified by immunostaining in one of the mice without treatment. In PF-2341066 treated mice, 4 of 7 mice showed LNCaP tumors in prostate tissues but no mouse had lymph node lesions.

To determine the effect of PF-2341066 on the growth of LNCaP cells in castrated mice, we carried out immunostaining of Ki67 on prostate tissues from the above different groups of mice. The Ki67 index from pre-castration mice was similar to the control mice treated with water, indicating development of castration resistant growth. The prostate tumor samples isolated from PF-2341066 treated mice showed a marked reduction in Ki67 staining (right, Fig. 4C) compared to the ones from control mice (left, Fig. 4C). The proliferative index as a percent of Ki67 positive cells was significantly lower from PF-2341066 treated mice than in control mice treated with water and pre-castration mice (Fig. 4D). Taken together, the above data suggested that the administration of a c-Met inhibitor after castration reduces subsequent proliferation of castration resistant prostate cancer cells.

## Discussion

Since the pioneering work in 1941 by Charles Huggins and Clarence Hodges that castration could be effective for metastatic prostate cancer, androgen deprivation therapy has been widely used to treat advanced prostate cancer [29]. Although different medications have been developed and applied to patients for the purpose of reducing the level of androgens or competitively repressing AR function, the fundamental premise of this therapy has remained almost unchanged. Within 2-3 years of the therapies, most patients develop more aggressive disease, CRPC. Therefore, it is urgent to develop more effective therapeutic strategies for advanced prostate cancer to prevent the development of CRPC.

Recent work including data from our lab has created a paradigm shift by showing a role of AR in transcriptional repression [11,30]. Our previous data demonstrated that AR suppressed c-Met transcription in LNCaP prostate cancer cells and castration of SCID mice bearing LNCaP xenografts induced c-Met expression in the tumors [11]. Knockdown of c-Met using specific c-Met siRNA inhibited the induction of c-Met expression by androgen depletion and repressed prostate cancer cell growth [31]. Decreasing *c-met *expression using ribozyme technology resulted in reduced prostate cancer cell proliferation and orthotopic tumor formation using PC-3 cells [26]. The c-Met receptor tyrosine kinase had been shown to be involved in tumor proliferation and progression and overexpression of c-Met had been associated with advanced prostate cancer [6-10]. These data suggested that the therapeutic strategy of inhibiting the activation of both HGF/c-Met and AR signaling pathways should be considered in the treatment of advanced prostate cancer.

In this study, we used two recently developed small molecule inhibitors for c-Met receptor, PHA-665752 and PF-2341066, to test the inhibition of c-Met activation in the growth and progression of prostate cancer cells. First, we evaluated the efficacy of two small molecule inhibitors for c-Met, PHA-665752 and PF-2341066, in a variety of prostate cancer cell lines. We observed significant inhibitory effects of these inhibitors on all the cell lines used here. In addition, we also confirmed that these small molecule inhibitors block phosphorylation of c-Met to inhibit its activation and downstream signalling [12,14]. In this study, we compared the repressive effect of these small molecule inhibitors in androgen sensitive and insensitive cell lines. Interestingly, we observed a more pronounced effect of these inhibitors on androgen-insensitive cells than androgen-sensitive cells. This correlated with previous publications showing that androgen-insensitive cells possessed higher cellular levels of c-Met [7,8,10,32,33]. As we have shown previously, although these androgen-insensitive cells remained AR positive, the repressive effect of AR on c-Met expression in these cells appeared diminished or reduced [11]. Multiple lines of evidence have suggested that action and regulation mediated by AR may be different in androgen-sensitive and insensitive status [34,35]. Therefore, further investigation of the regulatory mechanisms of AR in these different stages would be extremely important to understand the molecular basis of the switch between androgen-sensitive and insensitive during prostate cancer progression.

To examine whether co-targeting androgen and c-Met signaling pathways could effectively impede the progression of prostate cancer, we initially demonstrated the feasibility and effectiveness of the c-Met inhibitor on repression of the proliferation of prostate cancer cells in a xenograft mouse model using an orally bioavailable c-Met small molecule inhibitor, PF-2341066 [11,20,21]. We observed a significant 48% reduction in gross tumor weight and 45% reduction in proliferation when comparing PF-2341066 treated mice to control mice. Using an AR positive, androgen insensitive prostate cancer cell line, LNCaP C4-2, we showed for the first time that PF-2341066 inhibited the growth of AR positive prostate tumor xenografts *in vivo*.

Next, we tested targeted therapy to c-Met with castration in an orthotopic mouse model to determine the effectiveness of combination therapy in inhibiting prostate cancer progression. Intraprostatic injection of LNCaP cells into SCID mice had been shown to lead to tumor formation within one month in this mouse model [36-38]. Subsequent castration of these mice initially delayed tumor growth but LNCaP cells eventually regained growth ability and developed androgen-insensitive tumors [22-25]. We chose a dosing schedule of three weeks based upon a prior study showing *in vivo *effectiveness of PF-2341066 on prostate cancer cells [14]. Intriguingly, we observed a significant 85% reduction in proliferation when comparing PF-2341066 treated castrated mice versus control castrated mice. This elucidated that targeting of c-Met signalling as an adjunct to standard androgen deprivation therapy may reduce the development of castration resistant tumors. In addition to PHA-665752 and PF-2341066 tested in our study, other small molecule c-Met receptor tyrosine kinase inhibitors have also been developed and showed an inhibitory effect in non-prostate and prostate cancer cell lines [39,40]. In particular, Dai et al. reported in PC-3 cells that their c-Met inhibitor had anti-motogenic and anti-proliferative effects depending upon the drug concentration. This was similar to the previously published data on PHA-665752 and PF-2341066 [12,14] Taken together, these most recent findings and the results from this study provided important information on the potential of selective molecular therapy for advanced prostate cancers.

## Conclusions

Effective treatment for advanced prostate cancer has an urgent need in prostate cancer research. In this study, we demonstrated that two newly developed c-Met inhibitors, PHA-665752 and PF-2341066, repress the growth of a variety of prostate cancer cell lines. We observed an inhibitory effect of PF-2341066 on the proliferation of AR positive tumors in renal subcapsular mouse models. Moreover, using an orthotopic xenograft model, we showed that co-inhibition of c-Met and androgen signalling pathways using PF-2341066 and castration significantly reduced the proliferation of prostate cancer cells during progression after castration. Data from this study shed fresh light on possible future treatment by co-targeting c-Met and androgen signalling pathways in advanced prostate cancers.

## Competing interests

JGC is an employee of Pfizer. The other authors declare that they have no competing interests.

## Authors' contributions

ZS and JGC conceived of the study. WHT, CZ, and CC collected the data. WHT and ZS contributed to data analysis and wrote the manuscript. All authors read and approved the final manuscript.

## Pre-publication history

The pre-publication history for this paper can be accessed here:

http://www.biomedcentral.com/1471-2407/10/556/prepub
